# Genetic Characterization and Phylogenetic Analysis of *Babesia bigemina* Isolates in Cattle from South Africa Based on *BgRAP-1*, *BgAMA-1* and *BgβTUB* Genes

**DOI:** 10.3390/biology14040355

**Published:** 2025-03-28

**Authors:** Phillip Senzo Mtshali, Moses Sibusiso Mtshali

**Affiliations:** 1 Veterinary Parasitology Programme, Research and Scientific Services Department, National Zoological Gardens of South Africa, Pretoria 0001, South Africa; 2 School of Molecular and Life Sciences, University of Limpopo, Private Bag X 1106, Sovenga, Polokwane 0727, South Africa

**Keywords:** *Babesia bigemina*, cattle, South Africa, genetic characterization, phylogenies

## Abstract

*Babesia bigemina* is one of the most important etiological agents of bovine babesiosis, a tick-borne disease posing a major threat in the livestock industry globally, including South Africa. Despite the huge economic impact of cattle babesiosis in South Africa, antigenic variation observed among *B. bigemina* strains worldwide has impeded the successful development of a single vaccine with the potential to eliminate the disease. As such, there is still a dearth of information regarding the conservation of *B. bigemina* genes encoding functionally important proteins that play a crucial role during the invasion of bovine erythrocytes by merozoites. Fifty blood samples previously collected from cattle in eight provinces of South Africa were genetically tested for the presence of *B. bigemina* DNA fragments using four nested PCR-based assays. The genes targeted coded for *Spe*I-*Ava*I restriction fragment, rhoptry-associated protein 1 (*BgRAP-1*), apical membrane antigen 1 (*BgAMA-1*) and β-tubulin (*BgβTUB*). PCR-generated fragments of randomly selected samples were sequenced. BLAST searches in GenBank were performed with newly determined sequences to search for homologous sequences. Neighbor-joining phylogenies were inferred from aligned, contiguous sequences of *BgRAP-1*, *BgAMA-1* and *BgβTUB* genes. Nested PCR assays generated single fragments of 170 bp, 472 bp, 765 bp and 302 bp for *Spe*I-*Ava*I, *BgRAP-1*, *BgAMA-1* and *BgβTUB* fragments, respectively. Of the 50 bovine samples tested by nested PCR, 82% (42/50; 95% CI = 69.2–90.2%), 68% (34/50; 95% CI = 54.2–79.2%), 50% (25/50; 95% CI = 36.6–63.4%) and 46% (23/50; 95% CI = 33.0–59.6%) possessed *B. bigemina*-specific *Spe*I-*Ava*I, *BgRAP-1*, *BgAMA-1* and *BgβTUB* DNA fragments, respectively. The *BgRAP-1*, *BgAMA-1* and *BgβTUB* sequences of South African *B. bigemina* isolates shared 98–100% similarity with previously reported sequences of strains originating from cattle in countries other than South Africa. The high genetic conservation observed among geographical isolates of *B. bigemina* suggests the conserved functional role of BgRAP-1 and BgAMA-1 proteins as potential candidates that could be incorporated in recombinant subunit vaccines.

## Simple Summary

One of the main causative agents of bovine babesiosis, a tick-borne disease that poses a serious risk to the livestock industry worldwide, including in South Africa, is *Babesia bigemina*. Even though bovine babesiosis has a significant economic impact on South Africa, the successful development of a single vaccine with the potential to eradicate the disease has been hampered by antigenic variation identified among *B. bigemina* strains worldwide. The conservation of *B. bigemina* genes that encode functionally significant proteins that are essential for the merozoite invasion of bovine erythrocytes is thus still poorly understood. Four nested PCR-based tests were used to genetically analyze fifty blood samples that had previously been drawn from cattle in eight provinces in South Africa for the presence of *B. bigemina* DNA fragments. The substantial genetic conservation reported among regional isolates of *B. bigemina* implies the conserved functional role of BgRAP-1 and BgAMA-1 proteins as prospective candidates that might be integrated in recombinant subunit vaccines.

## 1. Introduction

Bovine babesiosis is known as an economically important infectious disease affecting cattle in tropical and subtropical regions of the world. This disease is induced by intraerythrocytic protozoan parasites of *Babesia* species [1]. *Babesia bovis* and *B. bigemina* are the main etiological agents of babesiosis, a tick-borne disease causing morbidity and mortality in cattle [2]. The acute phase of babesiosis is clinically manifested by anemia, hemoglobinuria, and fever in infected cattle [3]. Animals that survive primary infections become persistently infected for life and are reservoirs for tick transmission [4]. Although the disease caused by *B. bovis* is more pathogenic because of its clinical manifestations, *B. bigemina* infection also becomes severe if it goes untreated [5]. Of both parasites, *B. bigemina* has the highest prevalence because it is transmitted by several tick species, including *Rhipicephalus* (*Boophilus*) *microplus*, *R.* (*B.*) *decoloratus*, and *R. evertsi evertsi* [6,7]. This parasite has a complex life cycle, including an asexual stage while developing in the vertebrate host and sexual reproduction in the midgut of the tick host [4,8]. Asexual reproduction occurs inside erythrocytes where infected ticks introduce protozoans when feeding on mammalian host blood. The onset of infection takes place when babesial sporozoites enter red blood cells and multiply asexually. The sexual reproduction begins when ticks take up blood containing gametocytes [9].

Microscopic examination of Giemsa-stained blood smears has always been considered a gold standard for the diagnosis of acute babesial infections. However, the low sensitivity offered by microscopy techniques does not permit the detection of parasites in subclinical infections [10]. Alternatively, serology-based assays intended for detecting antibodies reactive with *Babesia* species have been described. The drawbacks associated with the use of serological tests include the occurrence of cross-reactions among *Babesia* species and the inconsistency in discriminating between previous exposure and current infections [11,12].

The advent of DNA-based assays has made it possible to accurately diagnose *B. bigemina* and *B. bovis* parasites in cattle due to the high sensitivity and specificity of these tests. The exploitation of these specific DNA-based tests to diagnose *Babesia* infections is of fundamental importance in the epidemiology of tick-borne diseases. Nested PCR tests specifically designed for targeting DNA of *B. bigemina* and *B. bovis* have been described by several authors [13,14,15,16,17,18,19,20,21]. Among these, Figueroa et al. [14] developed a nested PCR assay based on the *Spe*I-*Ava*I restriction fragment, which has been widely exploited for the diagnosis of *B. bigemina*. Another gene that has attracted considerable interest in the diagnosis of babesial infections is the rhoptry-associated protein-1a (*BgRAP-1*) gene, which is secreted by rhoptries organelles participating in the success of invasion and establishment of intracellular parasitic viability [22,23]. The latter gene is characterized by five copies organized in a single genomic region [21,24]. The *BgRAP-1* gene sequences of *B. bigemina* published in GenBank indicate that this gene is highly conserved among *B. bigemina* isolates originating from different countries across the globe; thus, this gene is considered a molecular marker and potent in identifying specific *B. bigemina* [25]. A similar observation was also the case for the *B. bigemina* gene coding for the apical membrane antigen (*BgAMA-1*); nucleotide sequences of the latter gene exhibit the highest degree of conservation among all strains sequenced to date, and their sequences were deposited in the GenBank repository [18,26]. Additionally, the β-tubulin (*BgβTUB*) gene has also been used in molecular diagnostics as a marker for species identification and discrimination in *Theileria* and *Babesia* parasites [27,28].

In South Africa, it is reported that the livestock industry accounts for up to 49% of the agricultural output [29], and that the tick-borne diseases in cattle are estimated to have an economic impact of USD 22 million per annum [30]. Therefore, it remains vital to establish the current status of the occurrence and geographical distribution of tick-borne parasites in cattle in order to implement efficient control strategies against cattle diseases. Of note, it has been established that the success in the development and application of recombinant vaccines against bovine babesiosis is hampered by antigenic variations observed in some parasite proteins [26]. These antigenic polymorphisms have been cited as an important route by which parasites evade the host immune response, resulting in disease outbreaks [31]. Currently, the use of acaricides and live attenuated vaccines are the only preventative measures used to control outbreaks [32]. As such, there is still a dearth of information regarding the conservation of *B. bigemina* genes encoding functionally important proteins that play a crucial role during the invasion of bovine erythrocytes by merozoites. It is reported in the literature that the conservation of babesial surface membrane proteins considered as potential candidate antigens may be critical for vaccine efficacy [33].

Therefore, given the huge impact of bovine babesiosis in the South African livestock industry and on the country’s economic security, the major impetus towards undertaking this study was to extend our current knowledge regarding the level and degree of sequence conservation among the field *B. bigemina* isolates of South African origin. In particular, the aim was to characterize the genes encoding rhoptry-associated protein 1 (*BgRAP-1*), apical membrane antigen 1 (*BgAMA-1*) and β-tubulin (*BgβTUB*) in *B. bigemina* isolates from field bovine samples. In addition, we also presented in silico bioinformatic analyses of sequences generated in this study against those from other countries published in GenBank.

## 2. Materials and Methods

### 2.1. Blood Samples

Fifty blood samples were randomly selected to validate nested PCR assays developed in this study. These samples form part of the sample collection of the Veterinary Parasitology Programme of the National Zoological Gardens of South Africa (NZG) and were previously collected from clinically healthy cattle across different locations in eight provinces of South Africa. Information on the vaccination status, age groups, husbandry practices and tick infestation status were not available at the time of sampling; other bovine blood samples were kindly collected and supplied by the farmers. The experimental collection of these samples was based upon the approval by the NZG Ethics and Scientific Committee. All blood samples were maintained at –20 °C in EDTA-coated vacutainer tubes.

### 2.2. DNA Isolation

DNA extraction from 200 μL of blood was performed using the ZR Genomic DNA^™^-Tissue MiniPrep kit (Inqaba Biotechnical Industries, Pretoria, South Africa) following the instructions of the manufacturer. Genomic DNA was eluted in 50 μL of elution buffer and stored at –20 °C until further analysis. DNA concentration was measured using NanoDrop^®^ ND-1000 (NanoDrop Technologies Inc., Wilmington, NC, USA).

### 2.3. Primer Design

Polymerase Chain Reaction and nested PCR primers targeting *BgRAP-1*, *BgAMA-1* and *BgβTUB* genes specific for *B. bigemina* were designed employing sequences of the corresponding genes published in GenBank. The accession numbers of *BgAMA-1* sequences extracted from GenBank were HM543726–HM543730 and JN572795–JN572801. The *BgRAP-1* sequences were available in GenBank under the accession numbers AF014757–AF014768. Accession numbers of the *BgβTUB* gene sequences used for primer design were AB634846, AJ289252, DQ104522 and EF060267. Primers were designed from conserved regions identified after creating multiple sequence alignments using the Clustal W algorithm embedded in BioEdit [34]. The species-specific primer sequences for the *Spe*I-*Ava*I nested PCR assay were obtained from the work published previously [14]. All the primers reflected in Table 1 were synthesized by Inqaba Biotechnical Industries.

### 2.4. PCR and Nested PCR Assays

Polymerase Chain Reaction and nested PCR assays to amplify *B. bigemina* species-specific *Spe*I-*Ava*I, *BgRAP-1*, *BgAMA-1* and *BgβTUB* fragments from field-derived bovine blood samples were performed using oligonucleotide primers listed in Table 1. In all PCR assays, the primary reaction mixture was composed of 5 μL of template DNA, 0.6 μM of each primer and 12.5 μL of DreamTaq Green PCR Master Mix (Inqaba Biotechnical Industries) in a final volume of 25 μL adjusted with nuclease-free water. The reactions were thermally cycled in a BIO-RAD T100^™^ Thermal Cycler (Bio-Rad Laboratories, Johannesburg, South Africa) under the following temperatures: initial denaturation at 94 °C for 3 min, followed by 25 cycles of denaturation at 94 °C for 30 s, annealing at 48.5–71.5 °C for 45 s and extension at 72 °C for 1 min. The final extension step was included at 72 °C for 5 min. The specific annealing temperatures are shown in Table 1.

For the secondary (nested) PCR, 1 μL of the first PCR product was used as the template. The composition of the nested PCR mixture was similar to that described above; except that nested PCR primers were used instead. The thermal cycling conditions were as follows: 94 °C for 3 min, followed by 94 °C for 30 s, 62–70 °C for 45 s and 72 °C for 1 min. The final extension at 72 °C for 10 min was included. For all PCR assays, a reaction with nuclease-free water instead of DNA was incorporated as a negative control.

Polymerase Chain Reaction products were resolved by electrophoresis on 1.5% (*w*/*v*) agarose gels containing 1× Biotium GelRed acid stain (Anatech Instruments, Johannesburg, South Africa). Gels were run for 30 min at 100 V in 1× TAE (Tris-acetate-EDTA) buffer, and DNA fragments were visualized under UV light. O’GeneRuler 1 kb DNA ladder (Inqaba Biotechnical Industries) served as the standard molecular weight marker.

### 2.5. Validation of Primers

The validity and specificity of new sets of primers were checked by performing BLASTDBv5 searches in GenBank. Purified DNA stocks of *B. bigemina*, *B. bovis*, *Theileria parva*, *Anaplasma centrale* and *Ehrlichia ruminantium* were employed to evaluate the specificity of all primer sets tested. DNA samples of *B. bigemina*, *B. bovis*, *A. centrale* and *E. ruminantium* were kindly supplied by Dr Nicola Collins (Department of Veterinary Tropical Diseases, University of Pretoria, South Africa), while *T. parva* came from Prof Oriel Thekisoe (Parasitology Research Programme, University of the Free State, South Africa). Reaction mixtures were prepared and cycled as described above using 2 μL of purified DNA stocks.

Due to the genetic relatedness between *B. bigemina* and *B. ovata* DNA sequences, we further subjected bovine samples to the PCR assay targeting a 504 bp *AMA-1* gene specific for *B. ovata*. The primer sequences and PCR conditions used were similar to those described in a recent study [35]. BovaF (5′-GAT ACG AGG CTG TCG GTA GC-3′) and BovaR (5′-AGT ATA GGT GAG CAT CAG TG-3′) were used as forward and reverse primers, respectively.

### 2.6. DNA Sequencing and Phylogenetic Analysis

Purified DNA fragments of selected samples, as shown in Table 2, were sequenced in both directions by Inqaba Biotechnical Industries using a Big Dye Terminator Kit (Applied Biosystems, Johannesburg, South Africa) with ABI 3130 XL Genetic Analyzer (Applied Biosystems). The determined sequences were aligned using the BioEdit software package for Windows 95/98/NT [34]. BLAST searches on the NCBI website were used to search for homologous sequences [36].

To construct phylogenetic trees, the sequences obtained in this study were aligned with the corresponding sequences published in GenBank and trimmed to equivalent lengths. Neighbor-joining trees were inferred using MEGA 5 software [37]. The Kimura two-parameter model [38] was used to estimate molecular distances, and bootstrapping analysis with 1000 replications was employed to determine the robustness of branches [39]. The EMBOSS needle program, version 6.3.1 (http://www.ebi.ac.uk/Tools/psa/emboss_needle/nucleotide.html, accessed on 7 March 2014) was used to perform pairwise comparisons of nucleotide sequences.

### 2.7. Nucleotide Sequence Accession Numbers

The nucleotide gene sequences reported in this study were deposited in GenBank under the following accession numbers: KF626581–KF626593 (*BgRAP-1*), KF626594–KF626606 (*BgAMA-1*) and KF626607–KF626618 (*BgβTUB*).

## 3. Results

### 3.1. Evaluation of Nested PCR Assays

Three diagnostic nested PCR assays developed in this study yielded single fragments with sizes corresponding to 470 bp for *BgRAP-1* gene, 765 bp for *BgAMA-1* gene and 302 bp for *BgβTUB* gene. The nested PCR assay based on the *Spe*I-*Ava*I restriction fragment presented an expected amplicon size of 170 bp. The results of PCR amplifications are presented in Table 2. While the PCR of *Spe*I-*Ava*I detected *B. bigemina* DNA in 41 out of 50 samples (82%; 95% CI = 69.2–90.2%), the PCR assays based on *BgRAP-1*, *BgAMA-1* and *BgβTUB* genes detected *B. bovis* in 34 (68%; 95% CI = 54.2–79.2%), 25 (50%; 95% CI = 36.6–63.4%) and 23 (46%; 95% CI = 33.0–59.6%) samples, respectively. Of the 50 samples tested, 22 (44%; 95% CI = 31.2–57.7%) possessed all four *B. bigemina* genes tested for. Only six samples did not yield PCR amplifications when subjected to any of the four nested PCR assays.

The primer specificity was tested by subjecting purified DNA samples of *B. bovis*, *T. parva*, *A. centrale* and *E. ruminantium* to nested PCR assays, which yielded no amplifications. In order to confirm the correct amplification of nested PCR fragments, randomly selected *Spe*I-*Ava*I amplicons from positive reactions were sequenced. The determined sequences were confirmed to correspond to the *Spe*I-*Ava*I restriction fragment of *B. bigemina* strains published in GenBank (accession nos. S45366 and FJ939724).

### 3.2. Comparative Sequence Analyses

In order to perform in silico analysis of nucleotide sequences of genes amplified by nested PCR primers developed in this study, positive field samples marked as “++” in Table 2 were selected for subsequent sequencing of *BgRAP-1*, *BgAMA-1* and *BgβTUB* genes.

From the analysis of *BgRAP-1* sequences, there were single nucleotides (SNPs) at 26 positions along the length of the sequenced fragments. Of these, 25 were synonymous between the sequences from FS-146, KZN-C24, GP-C7 and NW-C4. Nucleotide changes between sequences from FS-146, KZN-C24, GP-C7 and NW-C4 were synonymous with those of *B. bigemina* strains published in GenBank (M85187, AF014763 and AF014765) and shared identities ranging between 99.8 and 100%. On the other hand, the *BgRAP-1* sequences from DIVA, FS-189, MP-C1, MP-C11, MP-C12, WC-851, WC-10284, GP-C1 and GP-C19 were almost identical and shared 99.8–100% similarity with corresponding sequences published in GenBank (AF014757–AF014762).

Pairwise comparison of the *BgAMA-1* nucleotide sequences revealed a high degree of genetic conservation between the sequences determined in this study and those of *B. bigemina* strains available in GenBank. As indicated in Table 3, the highest sequence identities recorded ranged between 98.5 and 100%. The *BgAMA-1* sequences determined in this study shared 99.1–100% identity when compared to one another. A similar trend of sequence similarity (>98%) was recorded with GenBank strains of Mexican, Argentine and Italian origins. GenBank strains SP3 (JN572795) and M1P (JN572796) shared 99.6% sequence identity among them and revealed between 97.6 and 98.3% sequence identity with our sequences and other corresponding sequences published in GenBank.

Comparative analysis of the *BgβTUB* sequences indicated a high level of sequence conservation among South African *B. bigemina* isolates and GenBank strains. Nevertheless, there were SNPs observed along the length of the *BgβTUB* genes analyzed. For example, sequences from EC-19B, FS-146, GP-C1, KZN-C24, MP-C1 and MP-C11 were 100% identical and had nucleotide changes at only two positions. These sequences were also compared to *BgβTUB* sequences published in GenBank, revealing the maximum identity of 98.0–99.0% with strains Argentina (AB634846), Nigeria (AJ289252), Wayanad (EF060267) and Izatnagar (DQ104522). Similarly, the *BgβTUB* gene sequences from NW-C4, WC-851, GP-C19 and FS-189 shared 100% identity.

### 3.3. Analysis of Phylogenies

**Figure 1:**
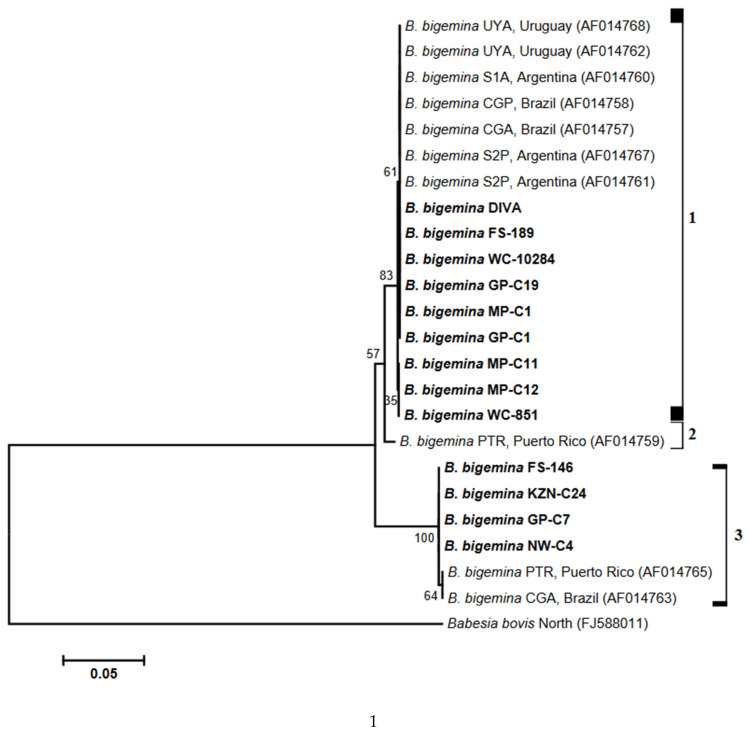
Phylogenetic tree constructed with the BgRAP-1 gene sequences from our *Babesia bigemina* isolates and GenBank strains. Nucleotide sequences determined in this study are shown in bold type, and accession numbers are given in parentheses. The horizontal scale bar indicates the number of base substitutions per site. Bootstrap values, computed as percentages of 1000 replicates, are indicated at branching points.

In studying the phylogenetic relationship among *B. bigemina* isolates, nucleotide sequences determined in this study were employed to infer neighbor-joining trees. Also incorporated in the phylogenies were sequences retrieved from GenBank. The phylogenetic tree constructed based on the *BgRAP-1* gene sequences grouped *B. bigemina* isolates into three clusters, with the sequence from the PTR strain (AF014759) forming its own cluster (Figure 1). The first cluster comprised sequences of nine South African *B. bigemina* isolates, grouping together with GenBank strains. In the third clade, four South African isolates formed a cluster with PTR (AF014765) and CGA (AF014763) strains.

**Figure 2:**
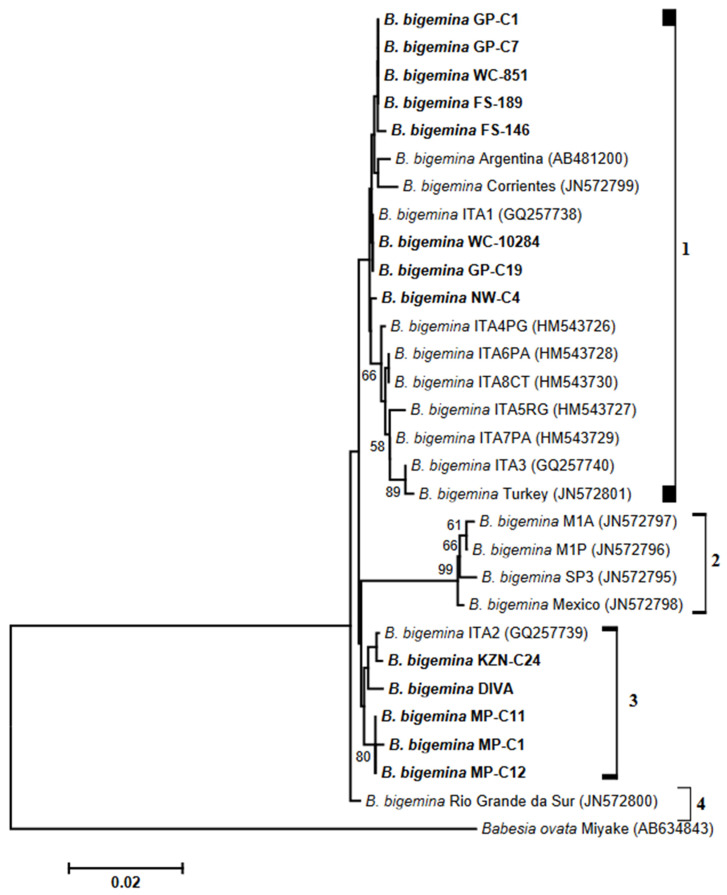
Neighbor-joining tree inferred with the BgAMA-1 gene sequences from our *Babesia bigemina* isolates and GenBank strains. Nucleotide sequences determined in this study are shown in bold type, and accession numbers are given in parentheses. The horizontal scale bar indicates the number of base substitutions per site. Bootstrap values, computed as percentages of 1000 replicates, are indicated at branching points.

The phylogeny based on *BgAMA-1* nucleotide sequences produced at least four clades (Figure 2). Sequences determined in the present study fell in clades 1 and 3, with a single sequence of Rio Grande da Sur (JN572800) found in cluster 4. Clades 1 and 3 comprised sequences derived from South African *B. bigemina* isolates, together with those retrieved from GenBank. Sequences in clade 3 were from Mexican *B. bigemina* isolates.

**Figure 3:**
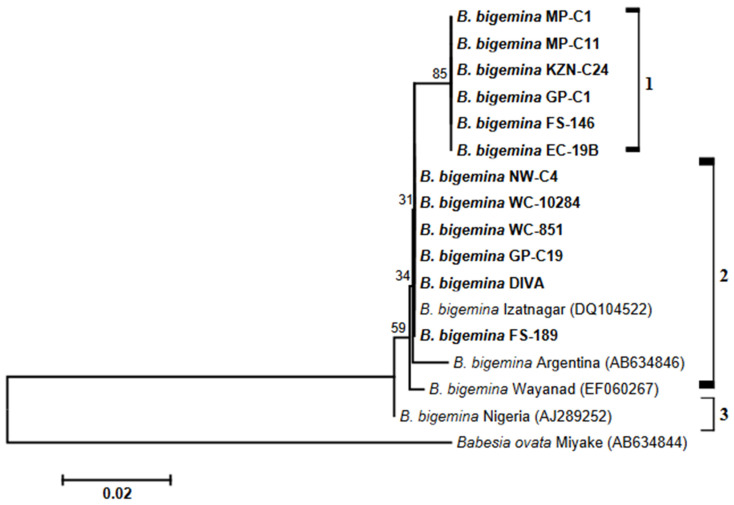
A phylogram created with *BgβTUB* nucleotide sequences from South African *Babesia bigemina* isolates and GenBank strains. Nucleotide sequences determined in this study are shown in bold type, and accession numbers are given in parentheses. The horizontal scale bar indicates the number of base substitutions per site. Bootstrap values, computed as percentages of 1000 replicates, are indicated at branching points.

Although there were only four *B. bigemina* sequences of *BgβTUB* genes retrieved from GenBank, the constructed phylogeny assembled *B. bigemina* isolates into three clusters (Figure 3). The first clade comprised exclusively sequences of *B. bigemina* isolates derived from South African bovine samples. GenBank sequences (DQ104522, AB634846 and EF060267) grouping with *B. bigemina* sequences determined in this study were found in clade 2. The *BgβTUB* sequence of the Nigeria strain (AJ289252) formed a monophyletic grouping in clade 3.

## 4. Discussion

The impact of bovine babesiosis worldwide has prompted many researchers to increase their research efforts in the search for effective vaccine candidates that could potentially confer absolute protection against infection by *Babesia* parasites. It must be acknowledged that, to date, there are currently no vaccines available to purge babesial infections in cattle globally. In essence, the success in the development of effective subunit vaccines against babesiosis is impeded by antigenic variations observed among heterologous *Babesia* strains. Eventually, this restricts the development of novel vaccine candidates based on antigens that are functionally vital for parasite growth and survival [5,40].

In South Africa, live attenuated strains of *Babesia* parasites have always been exploited as a means of immunizing cattle against babesiosis. However, the shortcoming associated with the use of live attenuated strains, which involves infection with bovine blood, relates to the possible cross-contamination with other blood-borne pathogens [41]. In addition, disease outbreaks emanating from vaccination with live attenuated strains have also been reported [42]. Therefore, it is vital to study the epidemiology of *Babesia* species and comprehend the degree of sequence variations among *Babesia* parasites, more specifically *B. bigemina*, in an attempt to develop effective control strategies against bovine babesiosis. In a recent study, we demonstrated that *B. bigemina* occurred more frequently in South African cattle than *B. bovis* [20].

In this study, we describe the successful application of three developed nested PCR assays for specifically detecting *B. bigemina* DNA fragments in field bovine samples. The *Spe*I-*Ava*I nested PCR-based assay [14] used as the control for the newly developed diagnostic assays detected *B. bigemina* DNA in more than 80% of field bovine samples tested. This was in contrast to *BgRAP-1*, *BgAMA-1* and *BgβTUB* assays, which amplified *B. bigemina* species-specific fragments in less than 70% of bovine samples examined, thus suggesting the high sensitivity of the *Spe*I-*Ava*I-based assay in diagnosing *B. bigemina*. These findings are in agreement with a previous study in which the *Spe*I-*Ava*I nested PCR assay detected more *B. bigemina*-positive samples than the *AMA-1* nested PCR assay [18]. However, it was discovered that *Spe*I-*Ava*I primers for the specific detection of *B. bigemina* also amplified the *Spe*I-*Ava*I-like fragment of *B. ovata* [18]. To further confirm this, we subjected the surveyed bovine samples to a *B. ovata*-specific PCR assay developed previously on the basis of the *AMA-1* gene [35]. As expected, no PCR amplifications of *B. ovata AMA-1* genes were observed. Until now, *B. ovata* has only been recorded in cattle from Mongolia, Japan, Korea, Thailand and China [43,44,45,46], and a recent study could not detect *B. ovata* DNA in bovine samples originating from South Africa [46].

Positive nested PCR products of *Spe*I-*Ava*I, *BgRAP-1*, *BgAMA-1* and *BgβTUB* fragments from selected samples were also sequenced to corroborate whether we amplified the correct target genes. The BLAST search in GenBank using newly determined nucleotide sequences confirmed that we amplified the correct genes. In order to rule out the possibility of *Taq* polymerase and sequencing errors, two independently derived nested PCR products from each isolate were sequenced. Indeed, no differences were found between the two sequenced fragments of each isolate. The obtained nucleotide gene sequences were also employed in silico sequence analysis and to study the phylogenetic relationship among *B. bigemina* isolates.

The ability of recombinant rhoptry-associated proteins (RAPs) to confer partial protective immunity against babesial infection with homologous and heterologous strains of *Babesia* species has stimulated interest among the researchers to identify both B-cell and T-cell epitopes [47]. These proteins are present on the surface of live merozoites and are considered important vaccine components as they are believed to play a vital role during the invasion of host cells [48]. In this study, we sequenced the *BgRAP-1* genes of *B. bigemina* isolates originating from South African bovine samples. In silico analysis of nucleotide sequences revealed that South African *B. bigemina* isolates are genetically similar to world strains. Although the genetic organization of the RAP-1 locus among babesial species is highly complex [49], the genetic conservation observed among *B. bigemina BgRAP-1* sequences determined in the present study may therefore be significant for vaccine efficacy. According to Giglioti et al. [21], the *BgRAP-1* gene of *B. bigemina* is characterized by five copies located in a single genomic region, and this could possibly explain why this gene was detected in some of the samples tested in this study (e.g., KZN-C31, MP-C2, MP-C19, WC-BC8, etc.) when other genes were not detected.

Apical membrane antigen 1 (AMA-1) is among the functionally important proteins employed by apicomplexan parasites to invade host cells. The well-studied AMA-1 protein is that of *Plasmodium falciparum*, an agent of malaria. Immune responses to *Plasmodium* AMA-1 are believed to have intense parasite inhibitory effects [50], suggesting that these proteins can be considered as important candidate antigens for vaccine development. As such, the AMA-1 protein of *B. bigemina* strains also appears to be essential during host cell invasion [51]. In the present work, although we observed SNPs occurring along the lengths of *BgAMA-1* genes of *B. bigemina* isolates examined, the high level of genetic conservation observed could imply that the AMA-1 protein of *B. bigemina* isolates merits inclusion in the development of subunit vaccines against babesiosis. It is also worth noting that this observation of genetic conservation among *B. bigemina BgAMA-1* genes is consistent with previous findings in which *BgAMA-1* nucleotide and amino acid sequences of Italian *B. bigemina* strains shared more than 99% identity with those of strains from Australia [26]. This feature of sequence conservation is commensurate with the proposed role of AMA-1 proteins as potential vaccine components.

The phylogeny created with *BgRAP-1* sequences grouped *B. bigemina* isolates into three clusters. The first cluster (clade 1) comprised South African isolates of *B. bigemina,* which appeared to be closely related to strains derived from Brazil, Argentina, Uruguay and Puerto Rico [52]. Among the South African isolates in clade 1, the sequences used for phylogeny construction originated from bovine samples collected at various geographical regions, including KwaZulu-Natal, Free State, Western Cape, Gauteng and Mpumalanga. Sequences of *B. bigemina* isolates in clade 3 were derived from samples collected in Eastern Cape, Free State, KwaZulu-Natal, Gauteng and Northwest; these sequences also clustered with those of strains from Brazil and Puerto Rico.

Phylogenetic analysis of *BgAMA-1* genes showed that micro-heterogeneities among *B. bigemina* isolates caused the formation of four clusters. Although the discriminatory power within cluster 1 was low, it was noteworthy that the sequences of isolates derived from samples collected at Gauteng, Western Cape, Free State and Northwest regions were phylogenetically related to those of *B. bigemina* strains from countries other than South Africa. Further, the *BgAMA-1* sequences of *B. bigemina* isolates from KwaZulu-Natal and Mpumalanga samples grouped with that of an Italian strain, sharing a sequence identity of 99.2–99.9%.

Sequences of the *BgβTUB* gene encoding the β-tubulin protein were also employed to infer a neighbor-joining tree. Phylogenetic analysis indicated that the *BgβTUB* sequences of South African *B. bigemina* isolates were found in two clades, and each clade comprised isolates derived from bovine samples collected at different geographical regions. Sequences of *B. bigemina* strains published in GenBank fell in the first clade. From in silico sequence analysis, it was also noted that there was a high degree of conservation between *BgβTUB* gene sequences of our isolates and those of GenBank strains. These findings are consistent with a previous study in which the coding sequence of the β-tubulin gene was found to be conserved among *Babesia* and *Theileria* species [27]. Nonetheless, observations of the phylogenetic clustering of *B. bigemina BgβTUB* sequences as demonstrated in this study may not be conclusive given that only four sequences could be retrieved from GenBank. In essence, the *BgβTUB* gene of *Babesia* species is considered an informative marker that is ideal for species identification and discrimination [27]. In addition, the β-tubulin genes of apicomplexan parasites are known to possess one or more introns [53,54]. While the first intron is conserved in all apicomplexan species studied so far, other introns of the β-tubulin gene are known to exhibit a great deal of variation both in length and in sequence, thus making this gene a suitable candidate as an informative marker [27].

## 5. Conclusions

We have described the successful application of three nested PCR assays that can be employed for the specific diagnosis of *B. bigemina* parasites in bovine samples. Sequences derived from positive nested PCR products provided more insights on the genetic conservation and phylogenetic relatedness among *B. bigemina* isolates of South African origin and those originating from other countries. Given that the occurrence of antigenic polymorphism among the functionally important surface proteins serves as the mechanism of parasite evasion from host immune response [16], it remains crucial to ascertain the degree of polymorphisms among representative *B. bigemina* isolates derived from geographically distinct regions. In particular, the high conservation of the *BgRAP-1* and *BgAMA-1* genes suggests that the proteins encoded by these genes could be suitable for inclusion in recombinant subunit vaccines for purging *Babesia* infections.

## Figures and Tables

**Table 1:** PCR and nested PCR primers used to amplify *Babesia bigemina* DNA fragments.

Target Gene	Assay	Primer Name	Primer Sequence (5′ → 3′)	Annealing	Product Size	Reference
*Spe*I-*Ava*I	PCR	BiIA	CATCTAATTTCTCTCCATACCCCTCC	55 °C	278 bp	[14]
		BiIB	CCTCGGCTTCAACTCTGATGCCAAAG			[14]
	nPCR	BiIAN	CGCAAGCCCAGCACGCCCCGGTGC	59 °C	170 bp	[14]
		BiIBN	CCGACCTGGATAGGCTGTGTGATG			[14]
*BgRAP-1*	PCR	BigRAP1	GTTATGTCAGCAGAGGTGGTTGGA	70.5 °C	564 bp	This study
		BigRAP2	ACCGAACAGGCGAGTTGTGAA			This study
	nPCR	BigRAP3	GAGGTTGTCAATGCTGAAATGGAAGC	71.5 °C	472 bp	This study
		BigRAP4	ACTTAGCCGCCGTAAAGTCAACG			This study
*BgAMA-1*	PCR	BigAmaF	CGTATGCCCACAGGAAAATGC	62 °C	1046 bp	This study
		BigAmaR	GTTTTCATGTTGAGAGCGGTGG			This study
	nPCR	BigAmaFN	CGGACTTCCTCGAACCGAT	66 °C	765 bp	This study
		BigAmaRN	CGTAGTTCGCCCAGTTCATACC			This study
*BgβTUB*	PCR	BtBigA	CTCTGACGAGCATGGAATCG	48.5 °C	408 bp	This study
		BtBigB	CTTTGGCCCAGTTGTTACCAG			This study
	nPCR	BtBigAN	CATGGCAGCCTGAAGCTTTG	67 °C	302 bp	This study
		BtBigBN	CGAAATTGTCGGGCCTGAAG			This study

**Table 2:** Results of nested PCR detection of *Babesia bigemina* DNA fragments in field samples.

Sample ID	Place of Origin	Collection Date	Source	Nested PCR Assay Results ^1^			
				*Spe*I-*Ava*I	*BgRAP-1*	*BgAMA-1*	*BgβTUB*
K0500-10	KwaZulu-Natal	May 2011	Bovine	+	+	+	+
KZN-C24	KwaZulu-Natal	May 2011	Bovine	+	++	++	++
KZN-C25	KwaZulu-Natal	May 2011	Bovine	–	+	–	+
KZN-C31	KwaZulu-Natal	May 2011	Bovine	+	+	–	–
KZN-C50	KwaZulu-Natal	May 2011	Bovine	+	+	+	–
KZN-C58	KwaZulu-Natal	May 2011	Bovine	+	+	+	+
KZN-C60	KwaZulu-Natal	May 2011	Bovine	+	–	–	–
ARUSHA	KwaZulu-Natal	May 2011	Bovine	+	+	+	+
COCO2	KwaZulu-Natal	May 2011	Bovine	+	–	–	–
DIVA	KwaZulu-Natal	May 2011	Bovine	+	++	++	++
NATALIE	KwaZulu-Natal	May 2011	Bovine	+	+	+	+
MP-C1	Mpumalanga	June 2011	Bovine	+	++	++	++
MP-C2	Mpumalanga	June 2011	Bovine	+	+	–	–
MP-C8	Mpumalanga	June 2011	Bovine	+	+	+	–
MP-C11	Mpumalanga	June 2011	Bovine	+	++	++	++
MP-C12	Mpumalanga	June 2011	Bovine	+	++	++	–
MP-C18	Mpumalanga	June 2011	Bovine	–	–	+	–
MP-C19	Mpumalanga	June 2011	Bovine	+	+	–	–
WC-723	Western Cape	July 2012	Bovine	+	+	+	+
WC-851	Western Cape	July 2012	Bovine	+	++	++	++
WC-10272	Western Cape	July 2012	Bovine	–	–	–	–
WC-10284	Western Cape	July 2012	Bovine	+	++	++	++
WC-11134	Western Cape	July 2012	Bovine	+	+	+	+
WC-BC8	Western Cape	July 2012	Bovine	+	+	–	–
FS-80	Free State	July 2005	Bovine	+	–	–	–
FS-156	Free State	July 2005	Bovine	+	+	+	+
FS-146	Free State	July 2005	Bovine	+	++	++	++
FS-189	Free State	July 2005	Bovine	+	++	++	++
FS-284	Free State	July 2005	Bovine	+	+	+	+
FS-289	Free State	July 2005	Bovine	+	–	–	–
GP-C1	Gauteng	December 2009	Bovine	+	++	++	++
GP-C2	Gauteng	May 2010	Bovine	–	+	–	–
GP-C3	Gauteng	May 2010	Bovine	+	+	–	–
GP-C7	Gauteng	March 2010	Bovine	+	++	++	+
GP-C9	Gauteng	May 2010	Bovine	+	–	–	–
GP-C19	Gauteng	May 2010	Bovine	+	++	++	++
EC-28A	Eastern Cape	May 2009	Bovine	+	+	–	–
EC-37A	Eastern Cape	May 2009	Bovine	–	–	–	–
EC-9B	Eastern Cape	May 2009	Bovine	+	–	–	–
EC-19B	Eastern Cape	May 2009	Bovine	+	+	–	++
NW-C2	Northwest	March 2011	Bovine	+	+	+	+
NW-C4	Northwest	March 2011	Bovine	+	++	++	++
NW-C7	Northwest	June 2012	Bovine	–	–	–	–
NW-C10	Northwest	June 2012	Bovine	+	+	–	–
NW-C17	Northwest	June 2012	Bovine	+	–	–	–
NC-1	Northern Cape	August 2012	Bovine	–	–	–	–
NC-8	Northern Cape	August 2012	Bovine	+	–	–	–
NC-20	Northern Cape	August 2012	Bovine	+	–	–	–
NC-24	Northern Cape	August 2012	Bovine	–	–	–	–
NC-41	Northern Cape	August 2012	Bovine	–	–	–	–

^1^ “(–)” denotes negative PCR amplifications; “(+)” denotes positive PCR amplifications; “(++)” denotes positive PCR amplicons selected for sequencing.

**Table 3:** Pairwise comparisons of *B. bigemina BgAMA-1* nucleotide sequences of South African isolates and GenBank strains.

Sequences ^1^		01	02	03	04	05	06	07	08	09	10	11	12	13	14	15	16	17	18	19	20	21	22
AB481200	01	**100**	99.6	99.2	99.1	99.3	99.1	99.5	99.1	98.9	99.1	98.9	99.1	99.1	98.9	99.5	99.6	99.7	99.7	99.6	99.7	99.7	99.6
GQ257738	02		**100**	99.3	99.2	99.7	99.2	99.6	99.5	99.1	99.2	99.3	99.5	99.5	99.3	99.9	99.7	99.9	99.9	100	99.9	99.9	100
GQ257739	03			**100**	99.1	99.1	99.1	98.9	99.3	98.9	99.9	99.2	99.3	99.3	99.5	99.5	99.3	99.5	99.5	99.3	99.5	99.5	99.3
GQ257740	04				**100**	99.5	99.5	98.8	98.7	99.9	98.9	98.8	98.9	98.9	98.8	99.3	99.2	99.3	99.3	99.2	99.3	99.3	99.2
HM543726	05					**100**	99.5	99.3	99.2	99.3	98.9	99.1	99.2	99.2	99.1	99.6	99.5	99.6	99.6	99.7	99.6	99.6	99.7
HM543727	06						**100**	98.8	98.7	99.3	98.9	98.8	98.9	98.9	98.8	99.3	99.2	99.3	99.3	99.2	99.3	99.3	99.2
JN572799	07							**100**	99.1	98.7	98.8	98.9	99.1	99.1	98.9	99.5	99.3	99.5	99.5	99.6	99.5	99.5	99.6
JN572800	08								**100**	98.5	99.5	99.3	99.5	99.5	99.6	99.3	99.2	99.3	99.3	99.5	99.3	99.3	99.5
JN572801	09									**100**	98.8	98.7	98.8	98.8	98.7	99.2	99.1	99.2	99.2	99.1	99.2	99.2	99.1
KZN-C24 *	10										**100**	99.3	99.5	99.5	99.6	99.3	99.2	99.3	99.3	99.2	99.3	99.3	99.2
MP-C1 *	11											**100**	99.9	99.9	99.5	99.5	99.1	99.2	99.2	99.3	99.2	99.2	99.3
MP-C11 *	12												**100**	100	99.6	99.6	99.2	99.3	99.3	99.5	99.3	99.3	99.5
MP-C12 *	13													**100**	99.6	99.6	99.2	99.3	99.3	99.5	99.3	99.3	99.5
DIVA *	14														**100**	99.5	99.1	99.2	99.2	99.3	99.2	99.2	99.3
NW-C4 *	15															**100**	99.6	99.7	99.7	99.9	99.7	99.7	99.9
FS-146 *	16																**100**	99.9	99.9	99.7	99.9	99.9	99.7
FS-189 *	17																	**100**	100	99.9	100	100	99.9
WC-851 *	18																		**100**	99.9	100	100	99.9
WC-10284 *	19																			**100**	99.9	99.9	100
GP-C1 *	20																				**100**	100	99.9
GP-C7 *	21																					**100**	99.9
GP-C19 *	22																						**100**

^1^ Accession numbers of sequences retrieved from GenBank (01 through 09) are given, and sequences determined in the present study are indicated with asterisks. The highlights is the standard way of representing data for this statistical test.

## Data Availability

The species-specific primers used in PCR assays were designed from multiple alignments of nucleotide gene sequences of *B. bigemina* strains retrieved from GenBank. All the nucleotide sequences from this study are publicly available on Genbank, and with the following accession numbers: KF626581, KF626582, KF626583, KF626584, KF626585, KF626586, KF626587, KF626588, KF626589, KF626590, KF626591, KF626592, KF626593, KF626594, KF626595, KF626596, KF626597, KF626598, KF626599, KF626600, KF626601, KF626602, KF626603, KF626604, KF626605, KF626606, KF626607, KF626608, KF626609, KF626610, KF626611, KF626612, KF626613, KF626614, KF626615, KF626616, KF626617, and KF626618.
